# Constitutive Endocytosis of the Neuronal Glutamate Transporter Excitatory Amino Acid Transporter-3 Requires ARFGAP1

**DOI:** 10.3389/fphys.2021.671034

**Published:** 2021-05-10

**Authors:** Kusumika Saha, Jae-Won Yang, Tina Hofmaier, SanthoshKannan Venkatesan, Thomas Steinkellner, Oliver Kudlacek, Sonja Sucic, Michael Freissmuth, Harald H. Sitte

**Affiliations:** ^1^Institute of Pharmacology, Center for Physiology and Pharmacology, Medical University of Vienna, Vienna, Austria; ^2^Institut Cochin, INSERM U1016, CNRS UMR8104, Université Paris Descartes, Paris, France

**Keywords:** excitatory amino acid transporter-3, endocytosis, regulated recycling, ARFGAP1, ARF6

## Abstract

The eukaryotic endocytic pathway regulates protein levels available at the plasma membrane by recycling them into specific endosomal compartments. ARFGAP1 is a component of the coat protein I (COPI) complex but it also plays a role in promoting adapter protein-2 (AP-2) mediated endocytosis. The excitatory amino acid transporter-3 (EAAT3) mediates the reuptake of glutamate from the synaptic cleft to achieve rapid termination of synaptic transmission at glutamatergic synapses. In this study, we identified two interacting proteins of EAAT3 by mass spectrometry (MS) ARFGAP1 and ARF6. We explored the role of ARFGAP1 and ARF6 in the endocytosis of EAAT3. Our data revealed that ARFGAP1 plays a role in the recycling of EAAT3, by utilizing its GTPase activating protein (GAP) activity and ARF6 acting as the substrate. ARFGAP1 promotes cargo sorting of EAAT3 *via* a single phenylalanine residue (F508) located at the C-terminus of the transporter. ARFGAP1-promoted AP-2 dependent endocytosis is abolished upon neutralizing F508. We utilized a heterologous expression system to identify an additional motif in the C-terminus of EAAT3 that regulates its endocytosis. Impairment in endocytosis did not affect somatodendritic targeting in cultured hippocampal neurons. Our findings support a model where endocytosis of EAAT3 is a multifactorial event regulated by ARFGAP1, occurring *via* the C-terminus of the transporter, and is the first study to examine the role of ARFGAP1 in the endocytosis of a transport protein.

## Introduction

Glutamate is taken up into cells by excitatory amino acid transporters (EAATs; Danbolt et al., 2016). These glutamate transporters are expressed predominantly in glial cells (EAAT1/SLC1A3, EAAT2/SLC1A2) and in specialized neurons (the cerebellar EAAT4/SLC1A6 and the retinal EAAT5/SLC1A7). Excitatory amino acid transporter-3 (EAAT3)/SLC1A1 (previously also referred to as the excitatory amino acid carrier-1, EAAC1) is found in neurons of the forebrain and in many non-neuronal tissues. In fact, the cDNA encoding EAAT3 was originally cloned from rat jejunum (Kanai and Hediger, 1992). Similarly, renal wasting of glutamate and aspartate is the most prominent phenotypic consequence resulting from genetic ablation of EAAT3 in mice (Peghini et al., 1997). This dicarboxylic aminoaciduria is also recapitulated by loss of function mutations in humans (Bailey et al., 2011). In the brain, the effects of EAAT3-deficiency are more subtle: in mice, there is evidence for enhanced age-dependent neuronal degeneration, which is attributed to enhanced vulnerability to oxidative stress due to glutathione depletion (Aoyama et al., 2008; Berman et al., 2011). Neurons, which cannot take up cysteine, depend on uptake of cysteine for maintaining glutathione levels and EAAT3 is one of the relevant neuronal cysteine transporters (Chen and Swanson, 2003; Himi et al., 2003). In contrast, there is little evidence for enhanced excitotoxicity in the absence of EAAT3: behavioral abnormalities are observed in young EAAT3(−/−) mice, i.e., reduced locomotor activity (Aoyama et al., 2006), but EAAT3(−/−) mice show less neuronal loss, when challenged with convulsant doses of pilocarpine (Lane et al., 2014). Similarly, one EAAT3-deficient individual showed signs of obsessive-compulsive disorder rather than seizures (Bailey et al., 2011). The fact that the absence of EAAT3 does not increase the susceptibility to seizures is in stark contrast to the phenotypic consequences of ablation of EAAT2 (Tanaka et al., 1997) or of EAAT1 (Watanabe et al., 1999). It can, however, be rationalized by taking into account the finding that EAAT3 only contributes to a very minor fraction of total forebrain glutamate (Holmseth et al., 2012). In addition, EAAT3 has been proposed to act primarily as a presynaptic glutamate buffer, limiting the activation of peri-/extrasynaptic NR2B-containing NMDA-receptors in CA1 pyramidal cells (Scimemi et al., 2009). Only a minor fraction of EAAT3 is at the cell surface, the vast majority is found within intracellular compartments of the neuronal soma and dendrites (He et al., 2000; Holmseth et al., 2012). The export of EAAT3 from the endoplasmic reticulum is subject to bidirectional regulation: it is enhanced by the ER protein reticulon RTN2B (Liu et al., 2008) and suppressed by the glutamate transporter associated interacting protein3-18 (GTRAP3-18; Lin et al., 2001; Ruggiero et al., 2008). In addition, EAAT3 undergoes rapid constitutive clathrin-dependent endocytosis and recycles *via* a Rab11-positive endosomal compartment (González et al., 2007). This shuttling of EAAT3 is compatible with one of its proposed functions, i.e., the buffering of presynaptic glutamate (Scimemi et al., 2009). In fact, many signals impinge on this cycle of endocytosis and exocytosis and thereby affect the surface levels of EAAT3 (Bjørn-Yoshimoto and Underhill, 2016). Internalization of EAAT2 has been reported to rely on the adapter protein arrestin-2/β-arrestin-1 (Ibáñez et al., 2016).

It is not clear, how EAAT3 is recruited into clathrin-coated pits. Here, we explored the hypothesis that the C-terminus of EAAT3 harbors sequence motifs, which support its constitutive internalization *via* the interaction with an adapter protein. We identified ARFGAP1 as this candidate adapter protein by showing that a disruption of this interaction reduced internalization of EAAT3.

## Materials and Methods

### Plasmids

The human EAAT3 was cloned into the pmGFP-C1 vector, using the following oligonucleotide primers to introduce unique flanking restriction sites (XhoI and EcoRI):

hEAAT3-Fw 5'GCGCCTCGAGCTGGGAAACCGGCGAGGA3'.
hEAAT3-Rv 5'GCGCGAATTCCTAGAACTGTGAGGTCTG 3'.

The point mutations in EAAT3 and human ARFGAP1 were generated using Quick Change II XL-site-directed mutagenesis kit (Stratagene Cloning Systems, La Jolla, CA, United States). All mutations were verified by automatic DNA sequencing.

Human His_6_-ARFGAP1 was used from our previous published work (Reiterer et al., 2008).

### Cell Culture and Transfection

Human embryonic kidney 293 (HEK293) cells were cultured and maintained in Dulbecco’s modified Eagle’s medium (DMEM), which was supplemented with 10% fetal bovine serum and antibiotics Penicillin-Streptomycin (Invitrogen, Carlsbad, CA, United States) at 37°C and 5% CO_2_. Cells were plated on tissue culture dishes (100 × 20 mm; Sarstedt, Nümbrecht, Germany) and transfected with jetPRIME® (Polypus Transfection) according to the manufacturer’s protocol. Transfected cells were analyzed 48 h post transfection. In all experiments, 2 μg DNA of the GFP-EAAT3 plasmid was used for transfections; the other constructs were co-transfected at variable ratios (indicated in the pertinent figure legends). The total DNA amount was kept constant by addition of empty vector (pBluescript).

### Preparation of Rat Brain Protein Extracts

Rats were dispatched by cervical dislocation and subsequently the brain was rapidly removed to prepare whole brain extracts. The brains were homogenized in ice-cold phosphate buffered saline (PBS) containing protease inhibitors (cOmplete®, Roche) and then centrifuged at 3,000 × *g* for 15 min. The resulting pellet was solubilized in lysis buffer containing 1% CHAPS, 50 mM Tris-HCl (pH 7.4), 0.5 mM EDTA, and a protease inhibitor cocktail on a rotator for 2 h at 4°C. After centrifugation at 10,000 × *g* for 30 min, the supernatant was collected for pull-down assay.

### Pull-Down Assay

GFP-tagged human EAAT3 (GFP-hEAAT3) and mGFP were transiently expressed in HEK293 cells and solubilized in lysis buffer [1% Triton X-100, 20 mM Tris-HCl (pH 8.0), 150 mM NaCl, 1 mM EDTA, 1 mM sodium orthovanadate, 5 mM NaF, 5 mM sodium pyrophosphate, and a protease inhibitor mixture] and subsequently immunoprecipitated with GFP-Trap beads (Chromotek, Planegg-Martinsried, Germany) overnight at 4°C. After four washes with lysis buffer containing 1% Triton X-100 and additional two washes with lysis buffer containing 1% CHAPS, EAAT3/beads and GFP/beads complexes were incubated with rat brain lysates (RBL; 8.65 mg) overnight at 4°C. To specify the interactions with RBL, analogous EAAT3/beads complex was incubated in 1% CHAPS lysis buffer without RBL at 4°C. After six washes, bound proteins were eluted in SDS sample buffer at 95°C for 3 min. Eluted proteins were size fractionated on sodium dodecyl sulfate polyacrylamide gel electrophoresis (SDS-PAGE) gels and visualized by a colloidal blue staining (Invitrogen).

### In-Gel Digestion

The protein bands were excised from SDS-PAGE gels, destained with 50% acetonitrile in 50 mM ammonium bicarbonate, and dried in a speed-vacuum concentrator. After reduction and alkylation of Cys, gel pieces were washed and dehydrated. Dried gel pieces were swollen with 25 mM ammonium bicarbonate (pH 8.0) containing 10 ng/μl trypsin (Promega) and incubated at 37°C for 18 h. Digested peptides were extracted with 50% acetonitrile in 5% formic acid (FA) and concentrated in a speed-vacuum concentrator (Yang et al., 2019).

### Liquid Chromatography-Coupled Tandem Mass Spectrometry

An electrospray ionization (ESI)-quadrupole-time-of-flight (QTOF; Compact, Bruker) coupled with an Ultimate 3,000 nano-HPLC system (Dionex) were used for liquid chromatography-coupled tandem mass spectrometry (LC-MS/MS) data acquisition. A PepMap100 C-18 trap column (300 μm × 5 mm) and PepMap100 C-18 analytic column (75 μm × 250 mm) were used for reverse phase (RP) chromatographic separation with a flow rate of 500 nl/min. The two buffers used for the RP chromatography were 0.1% FA/water and 0.08% FA/80% acetonitrile/water with gradient condition for 2 h. Eluted peptides were then directly sprayed into the mass spectrometer and the MS/MS spectra were interpreted with the Mascot search engine (version 2.4.1, Matrix Science, London, United Kingdom) against Swissprot database (559,077 sequences, released in January 2019) and the taxonomy was restricted to *Homo sapiens* (human; 20,414 sequences) for human EAAT3 from HEK293 cells as well as to rodentia (rodents; 26,759 sequences) for EAAT3-associated rat brain proteins. The search parameters were used with a mass tolerance of 10 ppm and an MS/MS tolerance of 0.1 Da. Carbamidomethylation on Cys, oxidation on Met were allowed with two missing cleavage sites and additionally phosphorylation on Ser/Thr was allowed for human EAAT3 protein searches. The Mascot cut-off score was set to 10 and proteins identified with two or more peptides were considered (Yang et al., 2019).

The mass spectrometry proteomics data have been deposited to the ProteomeXchange Consortium *via* the PRIDE (Perez-Riverol et al., 2019) partner repository with the dataset identifier PXD024524 and 10.6019/PXD024524.

### Uptake

The transfected cells were seeded onto poly-D-lysine coated 48-well plates (1 × 10^5^ cells/well). The uptake assay is performed 48 h post transfection; substrate uptake was determined as follows: The cells were gently washed with 0.5 ml/well of Krebs HEPES buffer (10 mM HEPES, 130 mM NaCl, 1.3 mM KH_2_PO_4_, 1.5 mM CaCl_2_, 0.5 mM MgSO_4_; at pH 7.4). Subsequently, the reaction was initiated by addition of 100 nM L-[^3^H] glutamate (specific activity = 50 mCi/mmol; Perkin Elmer, Boston, MA, United States) and allowed to proceed for 10 min at room temperature (23°C). In saturation experiments, the concentration of L-[^3^H] glutamate covered the range of 10–600 μM. For single point uptake, the final concentration of L-[^3^H] glutamate was 10 μM. Uptake was terminated by removing the substrate solution followed by a rapid wash with 0.5 ml/well of ice-cold Krebs HEPES buffer. The cells were subsequently lysed in 1% sodium dodecyl sulfate (0.5 ml/well) to release the cell-associated radioactivity, which was counted in the Packard 2300TR TriCarb Liquid Scintillation Analyzer. Non-specific uptake was determined in the presence of 100 μM L-trans-pyrrolidine-2,4-dicarboxylate (Sigma Aldrich, St. Louis, MO, United States).

### Immunoblotting

After two washes with ice-cold PBS, transfected cells were solubilized in RIPA buffer (10 mM Tris HCl, pH7.4, 150 mM NaCl, 1 mM EDTA, 1% Triton-X-100; 0.1% SDS) supplemented with protease inhibitors (cOmplete® protease inhibitor cocktail; Roche Applied Science, Indianapolis, IN, United States). Detergent solubilized proteins were denatured in electrophoresis sample buffer at 37°C for 15 min. The denatured protein samples were then subjected to denaturing polyacrylamide electrophoresis and transferred to nitrocellulose membrane. The membrane was then incubated for 16 h at 4°C with primary antibodies, i.e., a rabbit polyclonal antibody directed against GFP (1:2,000), a mouse monoclonal antibody against α-tubulin (1:5,000; Sigma Aldrich, St. Louis, MO, United States). After three washes, the membranes were incubated for 1 h at room temperature with secondary antibodies IRDye® 800 CW donkey anti-rabbit IgG and IRDye® 680 CW goat anti-mouse IgG both diluted 1:10,000 (LICOR, Nebraska, United States). Images of the membranes were captured by ODYSSEY® CLx Infrared Imaging system (LICOR, Nebraska, United States) and analyzed by ImageJ (NIH, United States).

### Cell Surface Biotinylation

Transfected cells were seeded into PDL-coated plates and allowed to adhere overnight. On the next day, the cells were washed thrice with ice-cold PBS^2+^ (PBS containing 1 mM MgCl_2_, 0.1 mM CaCl_2_) to remove FBS. The cells were then incubated twice with 1 mg/ml of sulfo-NHS-Biotin (Fisher Scientific, Waltham, MA, United States) in PBS^2+^ for 15 min in a shaker at 4°C. The reaction was quenched by two 15 min wash cycles with PBS^2+^ containing 100 mM glycine. Excess glycine was then removed by washing the cells in the PBS^2+^ buffer, and the biotinylated cells lysed in 0.3 ml RIPA buffer supplemented with protease inhibitors for 20 min on ice. The cell lysate was transferred into 1.5 ml Eppendorf tubes and the insoluble material removed by centrifugation at 10,000 × *g* for 10 min. The supernatant was collected and the protein concentration measured using the BCA method (Pierce, Rockford, IL, United States). An aliquot of the supernatant (100 μg) was incubated with streptavidin beads (30 μl of a preequilibrated 50% slurry: Thermo Scientific, Waltham, MA, United States) under head-over-tail rotation overnight at 4°C. On the next day, the beads were collected by centrifugation washed thrice with RIPA buffer to remove any unbound protein and the protein was then eluted from the beads by addition of denaturing sample buffer for 20 min at 37°C. Aliquots of the total lysates and of the affinity-purified material were resolved on denaturing polyacrylamide gels; the amount of EAAT3 was quantified by immunoblotting.

### GST Pull-Down Assay

The C-terminus of wild type EAAT3 (residues 467–524) and of a variant, in which F508 was replaced by alanine was fused to the carboxyl terminus of GST into the pGEX vector. The resulting fusion proteins – referred to as GST-C-terminus EAAT3-WT or GST-C-terminus EAAT3-F508A – were expressed in *Escherichia coli* BL21DE3 and purified from bacterial lysates by affinity chromatography on glutathione sepharose 4B (GE Healthcare, Buckinghamshire, United Kingdom) using 50 mM GSH to release the bound proteins. GSH was removed by repeated cycles of concentration and dilution in an Amicon pressure cell. Purified GST fusion proteins (30 μg) were incubated with cell lysates (100 μg) prepared from cells overexpressing ARFGAP1 in buffer containing 50 mM Tris HCl, pH 7.4, 150 mM NaCl, 0.1% Triton X-100. The reaction was carried out for 2 h on ice followed by addition of preequilibrated glutathione sepharose 4B beads (50 μl of 50% slurry) for 2 h in a rotor at 4°C. The beads were washed thrice with 1 ml buffer; the bound proteins were eluted after addition of sample buffer by denaturation for 5 min at 85°C. The eluates were analyzed by immunoblotting. The immunoreactive bands were detected with a mouse monoclonal antibody directed against hexahistidine (1:500; 3D5, Thermo Scientific, Waltham, MA, United States) and rabbit polyclonal anti GST antibody (1:1,000; Abcam, Cambridge, MA, United States) as described above.

### Confocal Microscopy

For confocal microscopy, 2.5 × 10^5^ transfected cells expressing GFP-tagged EAAT3, CFP-tagged ARFGAP1 or CFP-tagged ARF6 were seeded onto 22 mm glass bottom dishes (Invitro Scientific, Sunnyvale, CA, United States). The endoplasmic reticulum was visualized with the ER-Tracker™ Red dye (100 nM: Molecular Probes, Leiden, Netherlands) according to the manufacturer’s protocol. The plasma membrane was stained with trypan blue as described earlier (Korkhov et al., 2006). Images were captured Zeiss LSM780 equipped with an argon laser (at 30 milliwatts) and a 63x oil immersion objective (1.4 NA, Zeiss Plan-Neofluar). The images were processed using the Zen 2010 B SP1 software (Zeiss, Oberkochen, Germany).

### Primary Hippocampal Neurons and Immunocytochemistry

Hippocampi were isolated from 1‐ to 3-day old rat pups and cultures prepared as previously described (Montgomery et al., 2014). Briefly, the left and right hippocampi were dissected, digested in papain for 20 min at 37°C and subsequently triturated to dissociate the cells using increasingly smaller bore pipette tips. The cells were centrifuged for 4 min at 500 × *g* and resuspended in neuronal medium (Neurobasal A, 2% B27, 1% heat-inactivated calf serum, 0.4 mM glutamine, 50 μM kynurenic acid). The neurons were then seeded onto 29 mm glass bottom dishes with 20 mm bottom well (Invitro Scientific, Sunnyvale, CA, United States) pre-coated with poly-D-lysine. 5-Fluorodeoxyuridine was added to inhibit the proliferation of glia. After 7 days *in vitro*, the neurons were transfected with 2 μg DNA of the indicated plasmid using Lipofectamine2000 (Invitrogen Life Technologies, Waltham, MA, United States) following the manufacturer’s protocol.

After 24 h, the hippocampal neurons were washed twice with PBS and fixed with 4% paraformaldehyde for 15 min at room temperature. They were then permeabilized with PBS containing 0.1% Triton X-100 supplemented with 5% normal goat serum for 1 h at room temperature. Rabbit polyclonal MAP2 antibody (1:1,000) and mouse monoclonal vGlut1 antibody (1:500; both from Synaptic System Gottingen, Germany) were added to the permeabilization solution and incubated overnight at 4°C on a shaker. After three washes with PBS, the neurons were incubated with secondary antibodies (Alexa Fluor 568-goat anti rabbit IgG, and Alexa Fluor 405-goat anti mouse IgG, at 1:1,000 dilutions) for 1 h at room temperature. The samples were subsequently washed thrice with PBS and stored in PBS, at 4°C for imaging by confocal microscopy.

### Statistical Analysis

All data are presented as mean ± SD or SEM, where n indicates the number of experiments. Statistical analyses were performed using one-way ANOVA, followed by Dunnett’s or Sidak’s multiple comparison tests or by paired *t*-test as indicated in the figure legends.

## Results

### EAAT3 Associates With ARFGAP1 and ARF6

**Figure 1:**
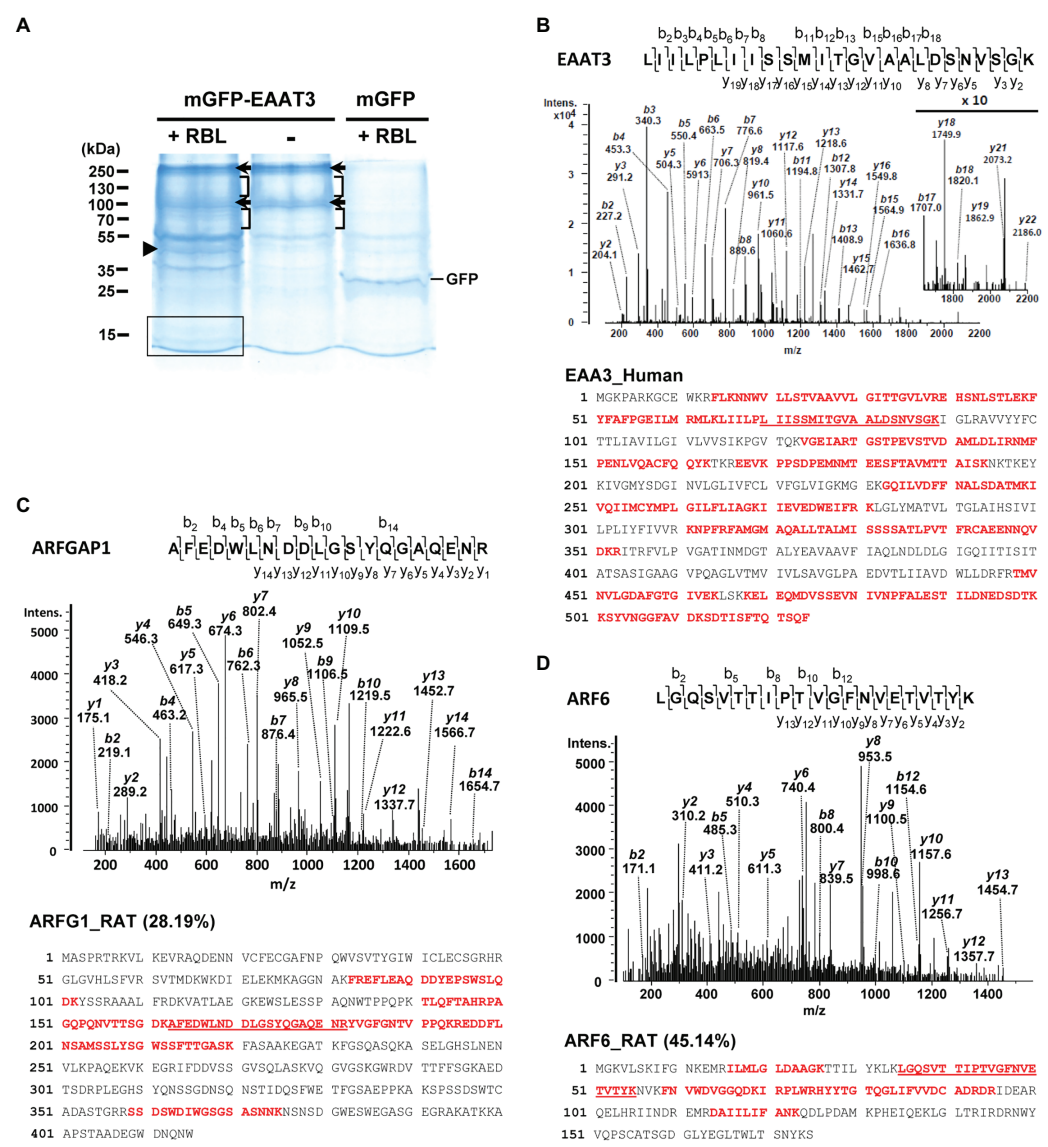
Identification of ARFGAP1 and ARF6 in the EAAT3 complex by LC-MS/MS. **(A)** Representative colloidal blue-stained sodium dodecyl sulfate polyacrylamide gel electrophoresis (SDS-PAGE) gel image with proteins pulled-down by EAAT3 and GFP proteins with (+) or without (−) rat brain lysates (RBL) incubation. Arrows and square brackets designate the bands and regions containing EAAT3 protein identified by LC-MS/MS. An arrowhead and a box indicate the band and region where MS identified ARFGAP1 and ARF6, respectively. The numbers refer to the positions of molecular weight markers. **(B)** (*top*) The double-charged (2^+^) peptide obtained at m/z 1266.24 was fragmented to produce a MS/MS spectrum with y-and b-ion series that identified the sequence LIILPLIISSMITGVAALDSNVSGK (amino acids 65–89) in human EAAT3 (*bottom*) Sequence coverage (59.16%) of EAAT3 (Uniprot ID: EAA3_Human) with identified peptides (bold) by MS/MS. **(C)** (*top*) The MS/MS spectrum, obtained at m/z 1165.02 (2^+^), for AFEDWLNDDLGSYQGAQENR (amino acids 163–182) of rat ARFGAP1. (*bottom*) Sequence coverage (28.19%) of ARFGAP1 (Uniprot ID: ARFG1_RAT) with identified peptides by MS/MS in the EAAT3-RBL complex. **(D)** (*top*) The MS/MS spectrum of ion at m/z 752.07 (2^+^) for the sequence LGQSVTTIPTVGFNVETVTYK (amino acids 35–55) of ARF6 in the EAAT3-RBL complex. (*bottom*) Sequence coverage (45.14%) of ARF6 (Uniprot ID: ARF6_RAT) with identified peptides by MS/MS.

To identify EAAT3 interacting proteins, we applied a proteomics approach based on LC-MS/MS for pulled-down EAAT3 binding proteins from detergent-solubilized RBL. GFP-hEAAT3 proteins were transiently expressed in HEK293 cells and immunoprecipitated by using anti-GFP antibody-conjugated beads (GFP-TRAP). These resulting GFP-hEAAT3/beads complexes were then incubated with and without 1% CHAPS-solubilized RBL (8.65 mg) to pull-down EAAT3-associated proteins. As a negative control, GFP/beads complexes were incubated with RBL and analyzed in parallel to specify GFP-hEAAT3-associated proteins. Pulled-down proteins were separated by SDS-PAGE and visualized by colloidal blue staining (Figure 1A). Several protein bands were exclusively presented or highly increased in the hEAAT3-engaged RBL sample compared to those in the samples of EAAT3 proteins without RBL incubation and GFP-RBL complexes (Figure 1A). Each lane of these three samples was excised into 10 pieces for in-gel tryptic digestion and the resulting tryptic peptides were subjected to LC-MS/MS. MS identified EAAT3 mainly from protein bands at about 250 and 85 kDa (Figure 1A, arrows) as well as from the regions containing 100–240 and 55–80 kDa (Figure 1A, square brackets) from the EAAT3 complex in the presence and absence of RBL incubation. These probably represent trimer and monomer forms of EAAT3 with mature and immature glycosylation based on the theoretical molecular weight of hEAAT3 (56.8 kDa) with GFP tag (26.9 kDa). Representatively, EAAT3 band at 250 kDa was identified with 796 peptides by MS/MS, which covered 59.16% of the human EAAT3 sequence (Figure 1B).

For the EAAT3-interacting proteins, MS identified ARFGAP1 and ARF6 with 16 and 9 peptides, respectively in the EAAT3-RBL complex (Figures 1C,D; Table 1). To specify their association with EAAT3, these results were compared with the identified proteins from GFP-RBL complexes. MS identified 1 peptide of ARFGAP1 and none of ARF6 peptide in the GFP complex (Table 1), indicating specific association of these proteins with EAAT3 proteins.

**Table 1:** Identification of ARF-GAP1 and ARF6 in the excitatory amino acid transporter-3 (EAAT3)-RBL complex by liquid chromatography-coupled tandem mass spectrometry (LC-MS/MS).

Uniprot ID^1^	Protein name	Residue	Peptide sequence	Mascot ions score^2^ (for each peptide)	
				GFP-EAAT3	GFP (negative control)
**ARFGAP1**					
ARFG1_RAT	ADP-ribosylation factor GTPase-activating protein 1	51–60	GLGVHLSFVR	31, 20	-
		83–102	FREFLEAQDDYEPSWSLQDK	36, 23, 15	-
		85–102	EFLEAQDDYEPSWSLQDK	23	-
		141–162	TLQFTAHRPAGQPQNVTTSGDK	59, 38	21
		163–182	AFEDWLNDDLGSYQGAQENR	52, 82	-
		183–195	YVGFGNTVPPQKR	29, 11	-
		196–220	EDDFLNSAMSSLYSGWSSFTTGASK	85, 74, 26, 45	-
**ARF6**					
ARF6_RAT	ADP-ribosylation factor 6	16–26	ILMLGLDAAGK	26	-
		35–55	LGQSVTTIPTVGFNVETVTYK	13, 40	-
		59–69	FNVWDVGGQDK	24, 38	-
		70–75	IRPLWR	10	-
		76–95	HYYTGTQGLIFVVDCADRDR	36, 56	-
		114–123	DAIILIFANK	10	-

1 http://www.uniprot.org/

2 The ions score for an MS/MS match is based on the calculated probability, P, that the observed match between the experimental data and the database sequence is a random event. The reported score is -10Log(P) (http://www.matrixscience.com/help/interpretation_help.html).

### A C-Terminal Motif Shapes the Endocytosis of EAAT3

**Figure 2:**
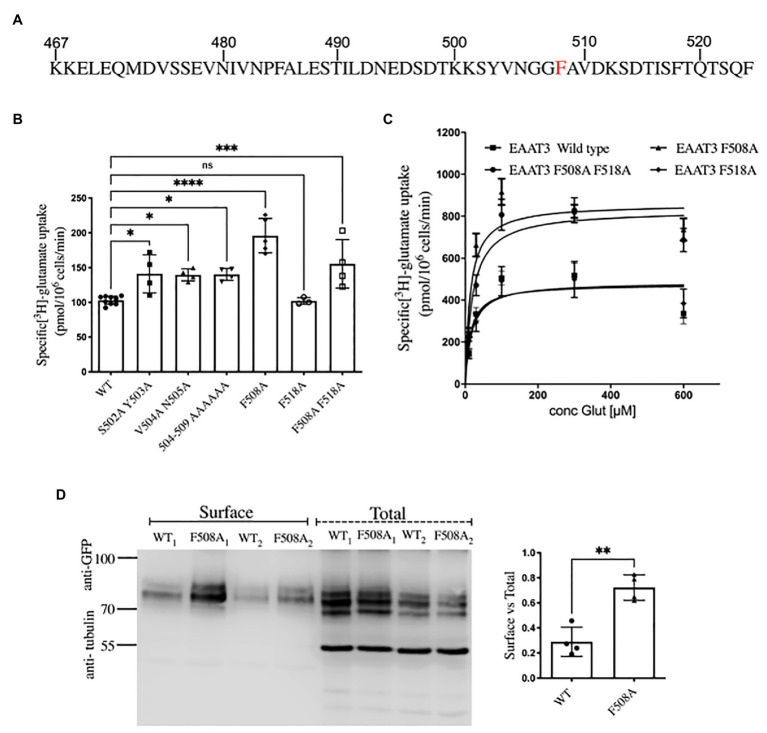
Surface levels of EAAT3 are determined by F508. **(A)** The amino acid sequence of the C-terminus of EAAT3 highlighting residue F508, which is required for constitutive endocytosis. **(B)** Uptake of L-[^3^H]glutamate by GFP-tagged EAAT3 and mutants. Data represents mean ± SD; (*n* = 4); ^****^p < 0.0001, one-way ANOVA followed by Dunnett’s multiple comparisons tests; ^****^p < 0.0001 between EAAT3-WT and EAAT3-F508A; ^*^p = 0.0129 between EAAT3-WT and EAAT3 S502A-Y503A; ^*^p = 0.0181 between EAAT3-WT and EAAT3-V504A-N505A; ^*^p = 0.0157 between EAAT3-WT and EAAT3-504-509 AAAAAA; *p* = 0.9999 between EAAT3 WT and EAAT3-F518A; ^***^p = 0.0005 between EAAT3-WT and EAAT3-F508A-F518A; ns-not significant. **(C)** Saturation curves for substrate uptake by GFP-tagged wild type EAAT3 (squares), EAAT3-F508A (triangles), EAAT3-F518A (circles), and the double mutant EAAT3-F508A-F518A (diamonds). The V_max_ values (in pmol/10^6^ cells/min) were: EAAT3 WT = 475.6 ± 34.5, EAAT3-F508A = 857.9 ± 45.7, EAAT3-F518A = 486.2 ± 48.3 and EAAT3-F508A-F518A = 829.4 ± 48.5. K_m_ were: EAAT3-WT = 14.0 ± 5.4, EAAT3-F508A = 13.7 ± 3.6, EAAT3-F518A = 17.8 ± 8.6, and EAAT3-F508A-F518A = 19.7 ± 5.8. The data represent mean values ± SEM (*n* = 4). **(D)** A representative Western blot from biotinylation assays, from two independent experiments denoted as WT_1_, F508_1_ and WT_2_, F508_2_. Left histogram shows the quantification of surface expression of GFP-EAAT3-WT vs. GFP-EAAT3-F508A. Band intensities of GFP-EAAT3 were calculated using Image J and normalized to α-tubulin. Data represents mean ± SD (*n* = 4; followed by unpaired *t*-test, ^**^p = 0.0014).

Having identified the interaction of EAAT3 with ARFGAP1 and ARF6, we wanted to examine how they contribute to trafficking of EAAT3. The C-terminus of EAAT3 (Figure 2A) contains sequence elements, which are known to determine trafficking of EAAT3: the stretch ^501^KSYVNGGFAVD^511^ specifies polarized localization in Madin-Darby canine kidney cells and somatodendritic targeting of EAAT3 in neurons (Cheng et al., 2002). The ^503^YVN^505^ motif has been implicated in regulated trafficking in a protein kinase C-dependent manner (Sheldon and Robinson, 2007). The last four amino acids (^520^TSQF^524^) represent a type I PDZ-binding motif, which has been shown to support the interaction with PDZK1/NHERF3 (Na^+^/H^+^-exchanger regulatory factor 3) and which counteracts endocytosis mediated by a tyrosine-based internalization signal ^503^YVNG^506^ (D’Amico et al., 2010). ARFGAP1 [the GTPase-activating protein of the ADP-ribosylation factor (ARF)] is required for AP-2-mediated endocytosis of the transferrin receptor (TfR); ARFGAP is recruited to the intracellular N-terminus of the TfR *via* two phenylalanine residues (F13 and F23) separated by 10 intervening residues (Bai et al., 2011). A similar arrangement, i.e., F508 and F518, is present in the C-terminus of EAAT3 (Figure 2A). We therefore surmised that these residues were important for constitutive internalization of EAAT3. Accordingly, we subjected the adjacent sequence of the C-terminus of EAAT3 to alanine-scanning mutagenesis. Only those transporters, which are at the cell surface, can mediate influx of [^3^H]glutamate. Hence, substrate uptake was determined to quantify surface levels of the resulting mutated versions of EAAT3. We used constructs, which were tagged with a GFP-moiety on the N-terminus. The addition of an N-terminal GFP-tag does not affect the trafficking of EAAT3 in neurons nor does it have any functional repercussions (Meera et al., 2005).

Replacement of the six amino acids in position 504–509 enhanced uptake as compared to WT. This stretch covers the previously identified internalization motif ^503^YVNG^506^ (D’Amico et al., 2010); accordingly, replacement of ^504^VN^505^ by alanine residues also enhanced uptake as compared to WT. However, sole substitution of F^508^ by alanine resulted in a mutant, which supported the highest level of substrate influx. In fact, uptake mediated by EAAT3-F508A exceeded that supported by EAAT3-S502A-Y503A. In contrast, the second phenylalanine residue EAAT3-F518A had no effect on the uptake as compared to WT and the double mutant EAAT3-F508A-F518A had no additive effect on the uptake compared to EAAT3-F508A (Figure 2B). If the surface levels of EAAT3-F508A were increased, maximum transport velocity was predicted to be enhanced. This was the case: the saturation experiments were performed which demonstrated that V_max_ of EAAT3-F508A and of the double mutant EAAT3-F508A-F518A were about 1.8-fold higher than that of wild type EAAT3 or of EAAT3-F518A. In contrast, the K_M_-values of all four variants of EAAT3 were comparable (Figure 2C).

**Figure 3:**
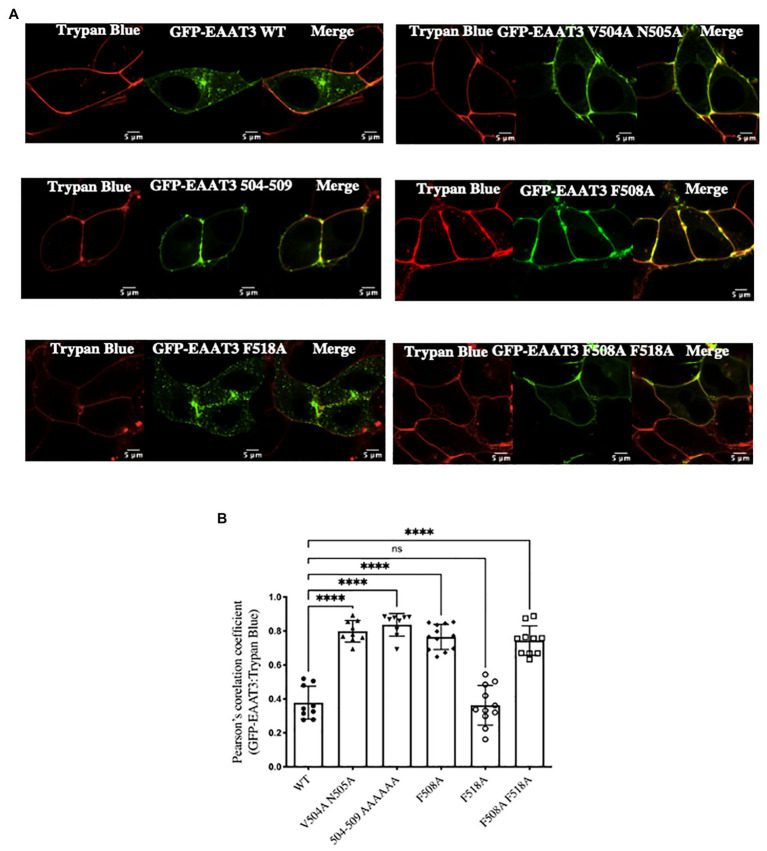
Visualization of EAAT3 mutants. **(A)** Representative confocal images of GFP-EAAT3 and different mutants thereof, with the cell surface visualized by trypan blue. Scale bar: 5 μm. **(B)** Pearson’s correlation coefficient between GFP-EAAT3 and Trypan Blue. Data represents mean ± SD; (*n* = 10–12 cells over three independent experiment); ^****^p < 0.0001, one-way ANOVA followed by Dunnett’s multiple comparisons tests; *p* = 0.9930 between EAAT3-WT and EAAT3-F518A; ns-not significant.

We confirmed that the increases in V_max_ corresponded to enhanced cell surface expression of the mutated transporter EAAT3-F508A by surface biotinylation experiments (Figure 2D): three major immunoreactive bands were seen in the total lysate; in contrast, the material retrieved after biotinylation of cell surface proteins contained only the upper two bands (Figure 2D). The lower band presumably corresponds to ER-resident core-glycosylated EAAT3. The ratio of this lower band to the upper bands was similar for wild type EAAT3 and EAAT3-F508A indicating that the mutation does not affect ER export. In contrast, the ratio of surface-biotinylated to the total EAAT3 immunoreactivity indicated that about 30% of wild type EAAT3 was on the cell surface, while essentially 70% of EAAT3-F508 was on the cell surface. Live cell confocal microscopy substantiated this finding (Figures 3A,B): EAAT3-F508A and EAAT3-F508A-F518A co-localized with the plasma membrane marker trypan blue. Their distribution was similar to that of the endocytosis-deficient mutants EAAT3-V504A and EAAT3-N505A, where the disrupted endocytic sorting motif resulted in confinement to the plasma membrane. The EAAT3-F518A, on the other hand, showed a pattern of distribution equivalent to that of the wild type transporter: there was a large intracellular pool, where the GFP-fluorescence accumulated in punctate structures (Figures 3A,B). Thus, our findings suggest that interfering with EAAT3-F508 impairs EAAT3 endocytosis and thereby increases the cell surface levels and transport activity of the transporter.

### ARFGAP1 Promotes Cargo Sorting of EAAT3 *via* the F508 Site

**Figure 4:**
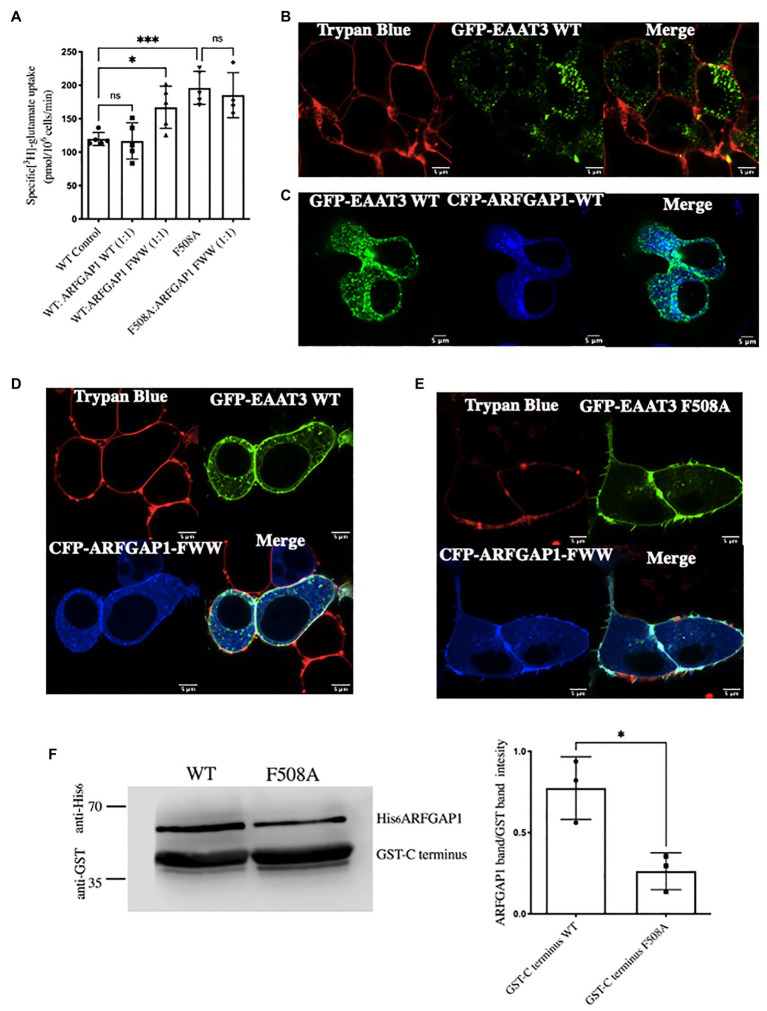
The role of ARFGAP1 in the endocytosis of EAAT3. **(A)** Single point uptake of GFP-EAAT3-WT and GFP-EAAT3-F508A co-expressed with CFP-ARFGAP1-WT or CFP-ARFGAP1-FWW in the ratio of 1:1. Data represents mean ± SD (*n* = 4);^***^p = 0.0002, one-way ANOVA followed by Sidak’s multiple comparison test; *p* = 0.9995 between EAAT3-WT Control and WT:ARFGAP1 WT(1:1); ^*^p = 0.0414 between EAAT3 WT Control and WT:ARFGAP1 FWW(1:1); ^***^p = 0.0008 between EAAT3 WT Control and EAAT3-F508A; *p* = 0.9587 between EAAT3-F508A and F508A:ARFGAP1 FW(1,1); ns-not significant. **(B)** Confocal image of GFP-EAAT3 WT depicting the intracellular EAAT3 pool. Scale bar: 5 μm. **(C)** Confocal image of GFP-EAAT3-WT co-transfected with CFP-ARFGAP1-WT depicting the intracellular EAAT3 pool. Scale bar: 5 μm. **(D)** Confocal image of GFP-EAAT3-WT and **(E)** GFP-EAAT3-F508A co-transfected with CFP-ARFGAP1 FWW denoting cell surface colocalization with trypan blue. Scale bar: 5 μm. **(F)** Representative western blot of pull-down assay with GST tagged C-terminus WT and C-terminus F508A with cell lysates from human embryonic kidney 293 (HEK293) transfected with His-ARFGAP1. Left histogram represents quantitative analysis of ARFGAP1 band intensity over GST C-terminus band. Band intensities of were calculated using ImageJ and data represents mean ± SD (*n* = 3; followed by paired *t*-test, ^*^p = 0.0164).

A triple mutant of ARFGAP1 (ARFGAP1-F332A-W366A-W385A – referred to as ARFGAP1-FWW) fails to facilitate AP2-mediated endocytosis of the transferrin receptor. This allows for selectively disrupting ARFGAP1 involvement in clathrin-mediated endocytosis, without influencing its activity within the coat protein I (COPI)-mediated anterograde trafficking pathway (Bai et al., 2011). We verified that ARFGAP1 played a role in the endocytosis of EAAT3 by relying on this ARFGAP1 mutant. Co-expression of EAAT3-WT with CFP-ARFGAP1-FWW, enhanced substrate uptake such that the transport rate was similar to that observed for the EAAT3-F508A mutant. In contrast, co-expression of CFP-ARFGAP1-FWW and EAAT3-F508A did not further enhance substrate influx (Figure 4A). We did not observe any significant change in EAAT3-WT mediated substrate uptake and distribution of the intracellular pool upon co-expression of ARFGAP1-WT (Figures 4A–C). We also examined the effect of CFP-ARFGAP1-FWW on the distribution of GFP-tagged wild type EAAT3 by confocal microscopy of live cells: the large intracellular pool of wild type EAAT3 (control cells shown in Figure 4B) or in the presence of CFP-ARFGAP1-WT (Figure 4C), was substantially reduced upon co-expression with CFP-ARFGAP1-FWW such that the bulk of EAAT3 colocalized with the membrane marker trypan blue (Figure 4D). In contrast, cell surface expression of EAAT3-F508A was not affected by CFP-ARFGAP1-FWW (Figure 4E). We explored whether there was a direct interaction between the C-terminus of EAAT3 and ARFGAP1. This was the case: the C-terminus of EAAT3 fused to GST pulled-down hexahistidine-tagged ARFGAP1 from cell lysates of HEK293. When the F508 was substituted by alanine, the resulting mutated C-terminus pulled-down substantially less ARFGAP1 (Figure 4F).

Adapter protein-2 enhances the GTPase activating protein (GAP) activity of ARFGAP toward ARF6. The GTPase-deficient mutant ARF6-Q67L interferes with the ability of ARFGAP1 to promote AP2-mediated endocytosis of the transferrin receptor (Bai et al., 2011). Our mass spectrometry data suggest that ARF6 is an interacting partner for EAAT3. We wanted to investigate if ARF6 was involved in the ARFGAP1 mediated endocytosis of EAAT3. Accordingly, we co-expressed ARF6-Q67L with EAAT3 to further confirm the role of ARFGAP1 in the endocytosis of EAAT3.

**Figure 5:**
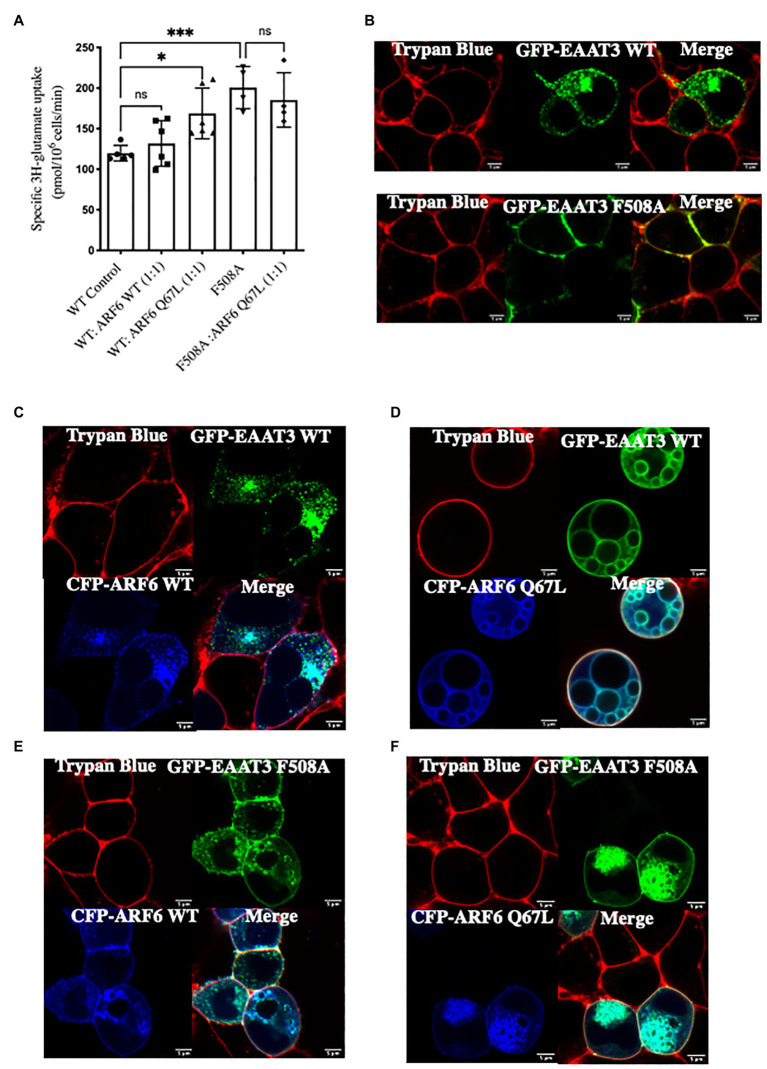
ARF6 acts a GTPase activating protein (GAP) substrate for ARFGAP1-mediated endocytic sorting. **(A)** Single point uptake of GFP-EAAT3-WT and GFP-EAAT3-F508A co-expressed with CFP-ARF6 WT or CFP-ARF6-Q67L in the ratio of 1:1. Data represents mean ± SD (*n* = 4); ^***^p = 0.0005, one way ANOVA followed by Sidak’s multiple comparisons test; *p* = 0.8517 between EAAT3 WT Control and WT:ARF6 WT(1:1); ^*^p = 0.0256 between EAAT3 WT Control and WT:ARF6 Q67L(1:1); ^***^p = 0.0010 between EAAT3 WT Control and EAAT3 F508A; *p* = 0.8977 between EAAT3 F508A and WT:ARF6 Q67L(1:1); ns-not significant. **(B–F)** Confocal image of GFP-EAAT3-WT or GFP-EAAT3-F508A cotransfected with CFP-ARF6-WT or CFP-ARF6-Q67L demonstrating the loss of endocytosis in the presence of CFP-ARF6-Q67L with no effect on GFP-EAAT3 F508A. Scale bar: 5 μm.

Glutamate uptake by EAAT3 was enhanced when cotransfected with ARF6-Q67L but not ARF6-WT. In contrast, the presence ARF6-Q67L did not affect substrate uptake in cells expressing EAAT3-F508A mutant (Figure 5A). Confocal microscopy also provided additional evidence that inhibiting ARF6 activity *via* ARF6-Q67L disrupted EAAT3 endocytosis without effecting EAAT3 F508A (Figures 5B–F). Wild type ARF6 is known to accumulate at the plasma membrane and in the endosomal compartment (Peters et al., 1995). Overexpression of ARF6-Q67L is known to cause deep plasma membrane invaginations in HEK293 cells (Peters et al., 1995) and accumulation of vacuoles in HeLa cells (Brown et al., 2001). Our confocal images are consistent with these phenotypes.

### Disruption of the COPI Machinery Impairs EAAT3 Anterograde Trafficking

**Figure 6:**
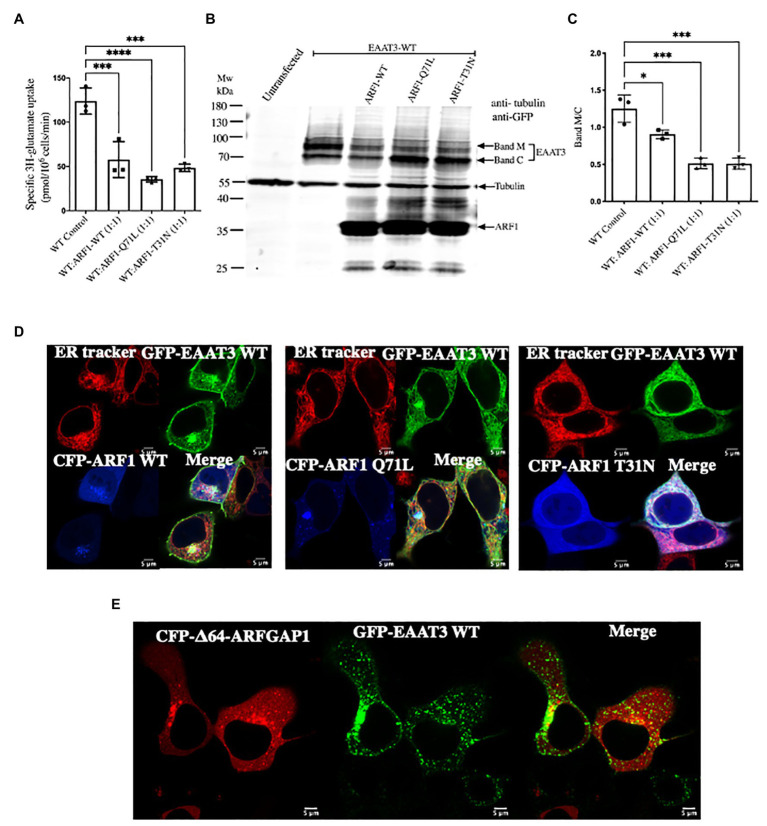
Arresting coat protein I (COPI) vesicle formation prevents EAAT3 anterograde trafficking. **(A)** Impairment of uptake by EAAT3 upon arresting COPI by co-transfecting with GFP-EAAT3 and CFP-ARF1 WT or CFP-ARF1-Q71L/T31N. Data represents mean ± SD (*n* = 4); ^***^p = 0.0001, one way ANOVA followed by Dunnett’s multiple comparisons test; ^***^p = 0.0006 between EAAT3 WT Control and WT: ARF1 WT (1:1); ^****^p < 0.0001 between EAAT3-WT Control and WT: ARF1 Q71L (1:1); ^***^p = 0.0003 between EAAT3-WT Control and WT: ARF1 T31N (1:1). **(B)** Representative western blot analysis prepared from whole cell lysate depicting the loss of glycosylated surface expression of EAAT3 upon co-expression of CFP-ARF1-Q71L/T31N. Band M represents the mature glycosylated form of EAAT3 and band C represents the core glycosylated form of EAAT3. It should be noted that the GFP antibody recognizes CFP, such that exogenously expressed CFP-ARF1 WT and CFP-ARF1-Q71L/T31N were also detected. **(C)** Analysis of the protein band intensities and comparison of glycosylated vs. ER fraction of EAAT3. Data represents mean ± SD (*n* = 3); ^****^p < 0.0001; one way ANOVA followed by Dunnett’s multiple comparisons test; ^*^p = 0.0124 between EAAT3-WT Control and WT: ARF1 WT (1:1); ^***^p = 0.0001 between EAAT3-WT Control and WT: ARF1 Q71L (1:1); ^***^p = 0.0001 between EAAT3 WT Control and WT: ARF1 T31N (1:1). **(D)** Confocal microscopy imaging of GFP-EAAT3 with CFP-ARF1 WT or CFP-ARF1 Q71L or CFP-ARF1-T31N shows restriction of EAAT3 within the ER upon co-expression of the ARF1 mutants. **(E)** Confocal microscopy imaging of GFP-EAAT3 with CFP-Δ64-ARFGAP1 depicting perinuclear punctate structure retention.

ARFGAP1 also supports anterograde trafficking of membrane proteins in COPI vesicles. Hence, it was essential to discern the role of the COPI-mediated anterograde pathway from endocytosis in EAAT3 trafficking. The assembly of the COPI coat and the subsequent fusion of COPI-coated vesicles with target membranes is contingent on the GTPase-cycle of ARF1: both, ARF1-Q71L and ARF1-T31N, which are trapped in the GTP‐ and the GDP-bound state respectively, disrupt anterograde trafficking in the secretory pathway (Dascher and Balch, 1994). There was a significant decrease in [^3^H] glutamate uptake, if cells co-expressed GFP-tagged EAAT3 and wild type CFP-tagged ARF1 or the mutants CFP-ARF1-Q71L and CFP-ARF1-T31N (Figure 6A). Immunoblotting of cellular lysates confirmed that this decrease in EAAT3-mediated transport was due to disruption in anterograde trafficking: overexpression of the ARF1 mutants reduced the mature glycosylated band and increased the core glycosylated band of EAAT3 (cf. bands M and C in Figure 6B) resulting in an altered ratio (Figure 6C). Overexpression of wild type ARF results in a stochiometric imbalance, which can also impede anterograde trafficking (Peters et al., 1995). Accordingly, both, CFP-tagged ARF1 and GFP-tagged EAAT3 accumulated in a perinuclear membrane compartment (Figure 6D, left hand set of images). In contrast, ARF1-T31N precludes delivery of COPI vesicles to the Golgi and thus traps cargo in the endoplasmic reticulum (Dascher and Balch, 1994). Accordingly, in cells expressing CFP-tagged ARF1-T31N, EAAT3 was confined to the endoplasmic reticulum (Figure 6D, right hand set of images). N-terminal truncation of ARFGAP1 results in its accumulation in punctate perinuclear structures, which presumably correspond to the ER-Golgi-intermediate compartment/ERGIC (Huber et al., 1998). In fact, Δ64-ARFGAP1 traps the GABA-transporter-1 (GAT1/SLC6A1) in the ERGIC (Reiterer et al., 2008). When co-expressed with CFP-tagged Δ64-ARFGAP1, EAAT3 was also retained in perinuclear punctate structures (Figure 6E).

### Loss of Endocytosis Does Not Disrupt Somatodendritic Targeting of EAAT3

**Figure 7:**
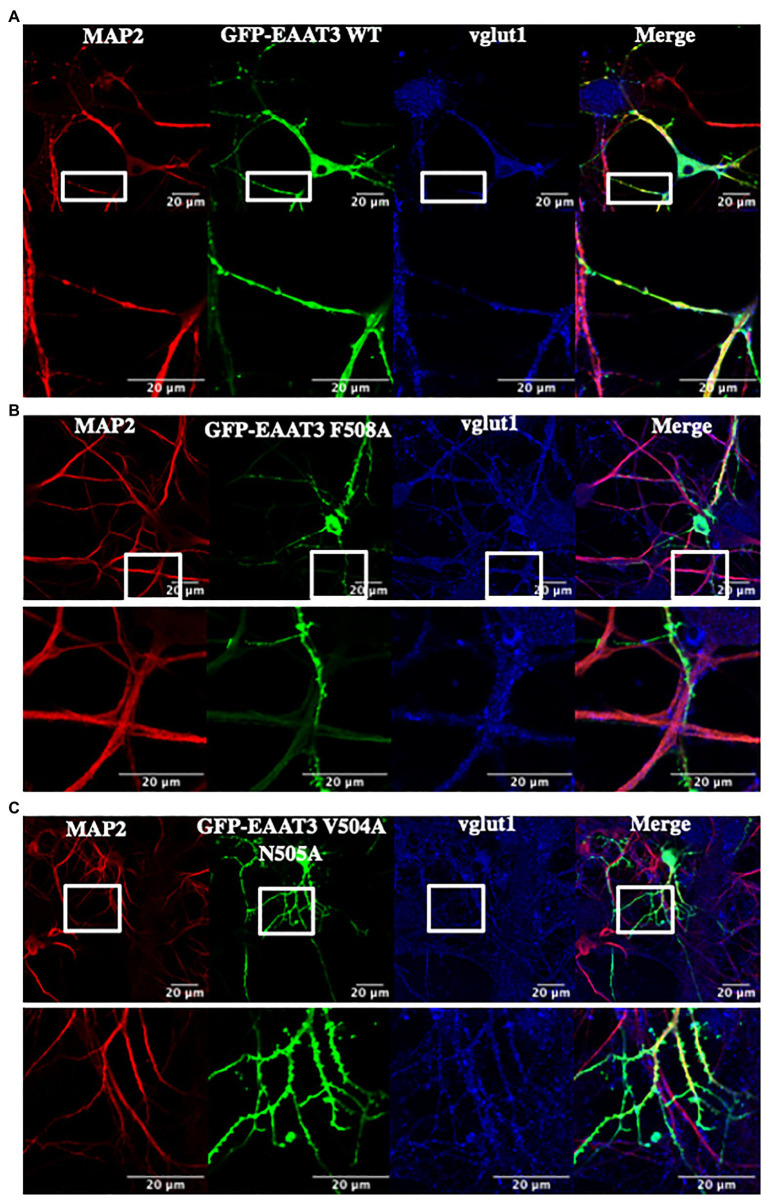
The role of endocytosis in the somatodendritic targeting of EAAT3. **(A)** *In vitro* transfection of cultured hippocampal neurons with 2 μg DNA encoding for GFP-EAAT3-WT or **(B)** GFP-EAAT3-F508A and **(C)** GFP-EAAT3-V504A-N505A shows correct cellular trafficking to the MAP2-positive compartments. The lower panel displays a higher magnification of the boxed region, revealing the presence of EAAT3 in the somato-dendritic regions of the neuron. Scale bar: 20 μm.

Taken together, these findings show that, in HEK293 cells, the role of ARF1 and ARFGAP1 in anterograde trafficking of EAAT3 can be discriminated from that of ARF6 and ARFGAP1 in endocytosis of EAAT3. However, HEK293 cells are not polarized. EAAT3 has a neuronal localization and is enriched in the hippocampus, cerebellum, and basal ganglia (Rothstein et al., 1994). EAAT3 is known to be enriched in the somatodendritic compartment in neurons (Cheng et al., 2002). We tested if endocytic sorting of EAAT3 *via* F508 would affect targeted delivery of EAAT3 in neurons. Hippocampal neurons were transfected with GFP-EAAT3 WT (Figure 7A), GFP-EAAT3 F508A (Figure 7B), or GFP-EAAT3 V504A N505A (Figure 7C). The vesicular glutamate transporter-1 (vGLUT1/SLC17A7) was used as a marker of glutamatergic neurons. The somatodendritic compartment was identified by staining for microtubule associated protein-2 (MAP2) as described (Montgomery et al., 2014). It is evident from both, the overviews and the magnified images shown in Figure 7 that the distribution of EAAT3 F508A did neither differ from that of wild type EAAT3 nor from EAAT3 V504A N505A: EAAT3 fluorescence colocalized with staining for MAP2. In addition, the magnified images showed clustering of all versions of EAAT3 on dendritic extensions. Hence, disruption of constitutive endocytosis did not affect neuronal distribution of EAAT3.

## Discussion

Clathrin-mediated endocytosis is considered the most important pathway for internalizing proteins from the cell surface (Bitsikas et al., 2014). This process is initiated by the formation of clathrin-coated pits, where the cargo proteins are trapped. Cargo recruitment is facilitated by AP-2 and other accessory proteins (Conner and Schmid, 2003; Maxfield and McGraw, 2004; Sorkin, 2004; Doherty and McMahon, 2009; McMahon and Boucrot, 2011). We found that ARFGAP1 promotes endocytic sorting of EAAT3 *via* the C-terminus residue F508. Abolishing cargo sorting by ARFGAP1 precludes internalization of the transporter, confining it to the cell surface. This effect is mediated *via* ARF6, acting as a GAP substrate for supporting AP2-mediated endocytosis. Moreover, inhibition of endocytosis does not impair somato-dendritic targeting of EAAT3 in primary neuronal cells. Our study reinforces the importance of C-terminus in the trafficking of EAAT3. The C-terminus has already been studied extensively for its contribution to the trafficking of EAAT3 (Cheng et al., 2002; Sheldon et al., 2006; D’Amico et al., 2010). Notably, residues ^501^KSYVNGGFAVD^511^ within the C-terminus have already been identified as important factors contributing to apical sorting in MDCK cells, as well as in dendritic sorting. This finding paved the way for studies of proteins that contribute to this process (Cheng et al., 2002). Our current study identified a specific interaction between residue F508 and ARFGAP1. This interaction is crucial to cargo sorting and AP2-mediated endocytosis. The substitution of F508 by alanine increases surface expression levels of EAAT3, without affecting the affinity of the transporter to its substrate.

Our work also provides a proof-of-principle for a direct interaction of EAAT3 C-terminus with ARFGAP1, which is appreciably decreased, but not completely abolished, for the F508A mutation. This resonates well with the fact that ARFGAP1 plays a role in both COPI and clathrin mediated endocytosis and that EAAT3-F508A can pass from Golgi to the cell surface. The ARFGAP1-FWW mutant fails to bind AP2 and clathrin, but interacts with β-COP (Bai et al., 2011). Consequently, this mutant in our study displays normal anterograde trafficking of EAAT3, while impairing its endocytosis.

It is important to note that we observed intact neuronal clustering of overexpressed EAAT3, despite defective endocytosis. This is evident for both, the ARFGAP1 interacting residue F508A and for the AP2-interacting residues (-YVN-). Dendritic targeting of EAAT3 as well as GluR1 and Kv4.2 is dependent on Myosin Va and the integrity of actin filaments in rat cortical neurons (Lewis et al., 2009). Additional investigations ought to address the question of precise molecular mechanisms promoting somato-dendritic targeting of EAAT3. Endocytosis of EAAT3 relies on the concurrence of two interactions: AP2 and ARFGAP1, the binding sites of which are in fact contiguous. It is plausible to predict that the binding is simultaneous, which is ought to be possible due to the trimeric nature of EAAT3. Precedent for that is the reported simultaneous binding of AP2 and PDZK1 (D’Amico et al., 2010).

Our study shows that ARF6 acts as a GAP substrate for ARFGAP1 to promote cargo sorting of EAAT3 during endocytosis. ARF6 has also been implicated in clathrin mediated endocytosis at the apical and the basolateral domain of polarized MDCK cells (D’Souza-Schorey and Chavrier, 2006). ARF6 binds and recruits NM23-H1, which supports dynamin-dependent vesicle fusion (Palacios et al., 2002). A previous study explored the effects of the psychoactive stimulant amphetamine, and showed that the endocytosis of EAAT3 occurs in a dynamin-dependent manner and is inhibited by dynasore, a cell permeable inhibitor of dynamin (Underhill et al., 2014). Our findings support and uphold the paradigm that multiple interlinked components of cell trafficking machinery orchestrate the surface density of EAAT3 proteins.

Clathrin-mediated endocytosis is a mode of endocytosis that internalizes molecules from the cell surface, thereby maintaining homeostatic regulation. This cellular event contributes to several physiological processes including intracellular signaling, nutrient uptake, cell adhesion, and migration. Several diverse proteins like TfR, low density lipoprotein receptor (LDLR), glucose transporter type-4, G-protein coupled receptors (GPCRs), and receptor Tyr-kinase (RTK), all follow such a cell cycle itinerary. However, the molecular process for sorting during endocytosis is as yet poorly understood (Maxfield and McGraw, 2004; Hsu et al., 2012; Robinson, 2015). EAAT3 is the first example of a multispanning transmembrane transporter protein that requires both ARFGAP1 and AP2 for endocytosis and may be relevant to many other transporters/multi-transmembrane domain containing proteins.

A previous study has identified numb, a clathrin associated sorting protein to be involved in the endocytosis of EAAT3 *via* the C-terminal YVNGGF motif (Su et al., 2016). Numb is an endocytic adaptor protein that localizes with endocytic organelles and associates with the α adaptin subunit of AP2 of the clathrin coated pits (Santolini et al., 2000). Numb has a N-terminal phosphotyrosine binding domain (PTB), C-terminal proline rich domain and an EH-domain. Numb binds its cargo *via* its PTB domain. In contrast other endocytic adaptor proteins like disabled (DAB) recognize non-phosphorylated NPXY internalization motifs *via* its N-terminal PTB domain (Yap and Winckler, 2015). Based on its mode of operation numb would require the tyrosine at position 503 (EAAT3-Y503) to be phosphorylated for its recognition of EAAT3 as a cargo. A study looking at the mechanism platelet derived growth factor (PDGF) stimulated trafficking of EAAT3 identified ^503^Y was not phosphorylated (Sims et al., 2000). Therefore, without a properly phosphorylated tyrosine side chain, it is difficult to envisage how numb should recognize the YVNGGF motif. Numb has also been implicated in negatively regulating membrane protrusion through ARF6 trafficking pathway by interacting with EFA6B a guanine nucleotide exchange factor (Zobel et al., 2018). Numb has also been shown to interact with EHD/Rme-1/Pincher family of endocytic proteins. These proteins are involved in recycling of plasma membrane proteins by clathrin mediated endocytosis and clathrin independent mechanism, which is regulated by Arf6 (Smith et al., 2004). It is tempting to speculate that numb plays a role in EAAT3 recycling and our studies in combination has identified different partners involved in endocytosis and recycling of EAAT3.

In conclusion, a dual regulatory signal in the C-terminus of EAAT3 denotes a firmly monitored process. By virtue of its eponymous action, ARFGAP1, apart from cargo sorting for endocytosis, generates a signal for the deactivation of ARF6. Thus, ARFGAP1-dependent internalization of EAAT3 is monitored by a signaling network, which allows for accurate control of internalized vs. surface bound transporter levels. This additional regulatory component of endocytosis of EAAT3 adds another advantage to the cellular machinery in regulating the physiological role of EAAT3.

## Data Availability Statement

The datasets presented in this study can be found in online repositories. The name of the repository and accession number can be found below: The European Molecular Biology Laboratory’s European Bioinformatics Institute (EBML-EBI) PRoteomics IDEntifications (PRIDE) Archive, https://www.ebi.ac.uk/pride/archive/projects/PXD024524.

## Ethics Statement

All animal experiments were conducted in accordance with protocols approved by the Animal Welfare Committee of the Medical University of Vienna and the Austrian Federal Ministry of Science and Research (License BMWF 66.009/0250-II/3b/2013).

## Author Contributions

KS, HS, J-WY, and MF have conceptualized the study. KS, J-WY, TH, SV, TS, and OK have performed the experiments. KS, HS, J-WY, MF, TS, OK, and SS have analyzed and interpreted the data. KS has written the draft of the manuscript and submitted it after obtaining input and approval from all other coauthors. All authors contributed to the article and approved the submitted version.

### Conflict of Interest

HS has received honoraria for lectures and consulting from AbbVie, Amgen, Astropharma, Astra Zeneca, Bano Healthcare, Chiesi, FOPI, Gebro, IIR, Lundbeck, MSD, Novartis, Pfizer, Roche, Rokitan, Sanofi-Aventis, Shire, and Vertex. MF has received honoraria for lectures and consulting from Amgen, Astra Zeneca, Astropharma, Baxter, Boehringer-Ingelheim, Celgene, Lundbeck, Merck-Sharp & Dohme, Novartis-Sandoz, Ratiopharm, and the Association of Austrian Sickness Funds.

The remaining authors declare that the research was conducted in the absence of any commercial or financial relationships that could be construed as a potential conflict of interest.
